# Host-Guest Inclusion Complexes between Amlodipine Enantiomers in the Biphasic Recognition Chiral Extraction System using Tartaric Acid and β-Cyclodextrin Derivatives as Positive Confirmation by using their Enantioselective Extraction

**DOI:** 10.3797/scipharm.1501-15

**Published:** 2015-06-22

**Authors:** Khaldun M. Al Azzam, Hassan H. Abdallah, Hairul N. Abdul Halim, Maizatul Akmam Ahmad, Hassan Shaibah

**Affiliations:** 1 Pharmacy program, Batterjee Medical College for Sciences and Technology (BMC), 21442 Jeddah, Kingdom of Saudi Arabia; 2 Chemistry Department, College of Education, Salahaddin University, Erbil, Iraq; 3 School of Bioprocess Engineering, Universiti Malaysia Perlis (UniMAP), Kompleks Pusat Pengajian Jejawi 3, 02600 Arau, Perlis, Malaysia

**Keywords:** Chiral drug, Enantioselective extraction, Racemic amlodipine, Biphasic recognition chiral extraction system, Computational calculations

## Abstract

The current work reports an extended theoretical study from our previous experimental work for the enantioselective extraction of amlodipine enantiomers in a biphasic recognition chiral extraction system (BRCES) consisting of hydrophobic D-diisopropyl tartrate dissolved in organic phase (*n*-decanol) and hydrophilic hydroxypropyl-β-cyclodextrin (HP-β-CD) in aqueous phase (acetate buffer) which preferentially recognize the *R*-enantiomer and *S*-enantiomer, respectively. The calculations were simulated using a semi-empirical PM3 method as a part of the Gaussian09 software package and were used to optimize the structures of the hosts, guests, and host-guest complexes in the gas phase without any restrictions. It was found that HP-β-CD has the strongest recognition ability among the three β-CD derivatives studied, namely HP-β-CD, hydroxyethyl-β-cyclodextrin (HE-β-CD), and methylated-β-cyclodextrin (Me-β-CD), due to the large interaction energies (E_comp_ = −14.3025 kcal/ mol), while D-diisopropyl tartrate has the strongest ability among the four tartaric acid derivatives studied namely; L-diisopropyl tartrate, D-diisopropyl tartrate, L-diethyl tartrate, and D-diethyl tartrate (E_comp_ = −5.9964 kcal/ mol). The computational calculations for the enantioselective partitioning of amlodipine enantiomers rationalized the reasons for the different behaviors for this extraction. The present theoretical results may be informative to scientists who are devoting themselves to developing models for their experimental parts or for enhancing the hydrophobic drug solubility in drug delivery systems.

## Introduction

Theoretical chemistry such as inclusion complexation with cyclodextrins (CDs) has been widely used as a powerful tool to obtain valuable insight into the mechanism and origin of enantioselectivity in several models. CDs are naturally-occurring cyclodextrins that are commercially available cyclic oligosaccharides containing 6, 7, 8-glucopyranose units and are referred to as α-, β-, and γ-CDs, respectively. Although the depth of the cavities for the three CDs is the same (~ 0.78 nm), their cavity diameters are ~ 0.57, 0.78, and 0.95 nm, respectively. A wonderful property of CDs is the ability to form inclusion complexes with a variety of small molecules of appropriate size via the influence of non-covalent interactions, e.g. hydrogen bonds, electrostatic, and van der Waals forces [1–3]. Moreover, CD inclusion complexation has been widely applied to many industrial branches such as pharmacy, foods, chemicals, and agriculture, etc. [3]. Therefore, the resultant inclusion complexes can induce modification of the physicochemical properties of the ‘guest’ molecules, particularly in solution stability and water solubility tests [2, 4–6]. Additionally, it has been reported that the inclusion complexation of drug molecules with CDs usually accompanied favorable changes in the physicochemical properties of the drug, such as solubility, dissolution rate, stability, and bioavailability, thus making them more suitable for oral drug delivery [7].

**Fig. 1:**
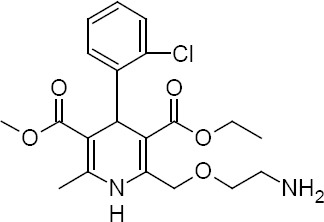
Structure of amlodipine (p*K*_a_ 8.6) [16, 17]

Amlodipine, 3-ethyl 5-methyl 2-[(2-aminoethoxy)methyl]-4-(2-chlorophenyl)-6-methyl-1,4-dihydropyridine-3,5-dicarboxylate (Fig. 1), is a racemic drug that belongs to the calcium channel blockers group, being used for treating hypertension and angina pectoris [8–10]. It acts as a calcium antagonist inhibiting the membrane influx of calcium ions in vascular smooth and cardiac muscles which in turn affects their contractile process and results in reduced blood pressure [11]. Although amlodipine is therapeutically used as a racemic drug, the vasodilating effect only resides in *S*-amlodipine. *R*-amlodipine is inactive, and is thought to be responsible for pedal edema through releasing nitric oxide in the peripheral blood vessels. Moreover, *S*-amlodipine is considered to be a more potent calcium channel blocker with about 2000 times the potency in an *in vitro* evaluation in the rat aorta compared to *R*-amlodipine [10, 12–15]. Thus, to reduce the incidence of peripheral edema and other side effects, it is beneficial to separate *R*-amlodipine from racemic (*R,S*)-amlodipine.

Nowadays, there is an increasing demand for enantiopure compounds due to the fact that enantiomers show different physiological effects on pharmacological activity, metabolism process, and toxicity on the human body. Thus, the development of new chiral technologies continues to be a very active area for scientists [18–21].

Tartaric acid derivatives are normal chiral extractants for many enantiomers that behave as a proton acceptor due to the oxygen atoms [18, 22]. Generally, separation factors using tartaric acid derivatives are under 1.2 [18, 23, 24]. This is attributed to the large number of transfer units required in the chiral liquid-liquid extraction process. Moreover, high separation factors may be achieved once used with crown ethers, but the extraction process would be very costly and toxic [25].

Recently, the chiral ligand-exchange model has been applied to liquid–liquid extraction technology and achieved high separation factors that are advantageous over chiral ligand-exchange chromatography especially for large-scale applications [26]. Therefore, tartaric acid derivatives and CDs have been used to discriminate amlodipine enantiomers in our previous work [27].

In 1995, several researchers focused on the study of inclusion complexes of CDs by semi-empirical methods, namely AM1 and PM3 to gain electronic properties and to have more information about the geometry of the complex formed. The obtained results suggested that PM3 should be more advantageous than AM1 and led to coinciding results which are in agreement with the experimental observations [28–32].

Later on, some studies were conducted regarding the performance of AM1 and PM3 calculation results for some model compounds such as hydroxyethyl ether and α-(1-4)-glucobiose. The obtained results revealed that PM3 is more advantageous than AM1 in CD inclusion processes because PM3 can predict the O–H…O hydrogen bonds better than the AM1 method [28, 33]. Upon direct structure optimization of α and β-CD with AM1 and PM3, AM1 gave badly distorted geometries due to unreasonable hydrogen bonding, whereas PM3 reproduced the crystalline structures rather well [28].

Recently, there has been increased interest in molecular modeling studies on the formation and stability of inclusion complexes of CDs with a variety of candidate drugs and other aspects of supramolecular chemistry. Several theoretical approaches have also been applied in these studies, for instance molecular mechanics, molecular dynamics, semiempirical methods, and the hybrid techniques (quantum-mechanics–molecular mechanics (QM–MM)) techniques [34–38]. Additionally, Hartree–Fock (HF) and particularly density functional theory (DFT) calculations have also been used reliably to describe host-guest interactions of CDs with several molecules [39, 40]. Although, of the rapid development and use of *ab*-initio and DFT, semiempirical methods still attract a great deal of attention due to their less computational demands. Semiempirical methods such as PM3 and PM6 have been found to give more accurate estimates of molecular properties when compared to HF and DFT methods, owing to the lower computational cost. Moreover, the use of calculation methods such as *ab*-initio or DFT on the totality of the inclusion complex will be computationally time-expensive because some systems may consist of many atoms such as 194 atoms as prescribed in the work of Attoui and Khatmi [41]. Therefore, the semiempirical methods described earlier appear as a promising field as it is not only used to describe the inclusion complexes [42], but also to predict the branched alkyl compounds or for the calculation of zinc complexes as prescribed by the work of Sierra and Kaya [43] and G. Frison and Ohanessian [44], respectively.

In the current paper, we have investigated the inclusion processes of amlodipine with β-CD and tartaric acid derivatives using the PM3 method in order to get insight into the conformation of this complex, and thus have a better explanation for the enantioselective extraction that occurred in our previous work using the biphasic recognition chiral extraction system (BRCES). Furthermore, to investigate and predict the energy of interaction (E_comp_) of the complexes between amlodipine/β-CD and amlodipine/tartaric acid derivatives, molecular mechanics methods were used with Autodock. Additionally, the obtained structures were further optimized by the semi-empirical PM3 method to obtain the binding energies of the studied inclusion complexes.

## Experimental

### Computational Method

The Gaussian09 [45] and GaussView 5.0.8 programs were used to run all the calculations in this study. Pop and his coworkers found the crystal structure of the complex of β-CD and mefenamic acid [46]. In order to achieve a high level of accuracy and reality, the crystal structure found by Pop was used as a starting structure to build the derivatives of β-CD. Three different types of β-CD were built, namely HP-β-CD (degree of substitution 0.5–1.3), HE-β-CD (degree of substitution 0.7), and Me-β-CD (degree of substitution 1.6–1.9). GaussView was used to visualize the crystal structure found by Pop and to build the three types of the β-CD by adding methyl, hydroxyl ethyl, and hydroxyl propyl groups to get Me-β-CD, HE-β-CD, and HP-β-CD, respectively. Producing the different types of β-CD was followed by minimizing energy in order to let the structure relax and to avoid any overlap between atoms using the molecular mechanics method that was implemented in the Gaussian09 program. In order to find the optimized energy of the β-CD derivatives, the semi-empirical PM3 method was used to optimize the structures and to calculate the ground state energy. Nowadays, it is well-known that the semi-empirical methods have proven to be an important tool for theoretical study of the CD inclusion complexes. Moreover, it allows a more realistic model with full conformational flexibility of supramolecular host-guest complexes [3]. The amlodipine molecule, in addition to the four isomers of the tartrate, namely D- and L-diisopropyl tartrate and D- and L-diethyl tartrate, were built using GaussView and then optimized with the PM3 semi-empirical method in the gas phase without any restrictions. The host-guest complexes of the amlodipine molecule with the derivatives of β-CD and the amlodipine with the isomers of tartrate derivatives were built using GaussView by putting the host and guest molecules online and close to each other and letting the complex relax using molecular mechanics, then optimizing the final complex with the PM3 method in order to get the energy of the complex. The binding energy or the complexation energy was calculated by Eq. 1:


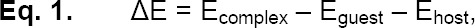


where E_guest_ is the energy of amlodipine, E_host_ is the energy of the β-CD derivative or the tartrate isomer, and E_complex_ is the energy of the host-guest complex.

### Materials and Chemicals

Racemic amlodipine was purchased from MTT Pharma in China (> 98%). β-CD derivatives, tartaric acid derivatives, and all solvents used were obtained from Sigma–Aldrich (St. Louis, USA). Deionized water was produced using a Milli-Q system (Millipore, Bedford, USA) and was used throughout for the preparation of solutions.

### Preparation of Aqueous and Organic Solutions

An aqueous solution of concentration 0.05 mmol/L racemic amlodipine and 0.10 mol/L β-CD was prepared in 10 mmol/L acetate buffer. The pH of the solution was adjusted to pH 4.5. On the other hand, the organic phase which consisted of 0.20 mol/L tartaric acid derivatives was dissolved in organic solvent.

### Extraction Procedure

The extraction procedure was conducted as prescribed in our previous work [27]. Equal volumes (each 3 mL) of aqueous solution containing 0.05 mmol/L of racemic amlodipine and 0.1 mol/L β-CD organic phase containing 0.20 mol/L tartaric acid derivatives was transferred into a 100 mL baffled flask. The flask was then shaken using a shaker for the desired contact time to reach the maximum extract of *R*-amlodipine in the organic phase (6 h). The experiment was carried out at different temperatures ranging from 5–30°C. In order to separate the two phases, the content of the flask was then transferred into a separation funnel. The concentrations of *R*- and *S*-amlodipine in aqueous phase were quantified by an HPLC system.

### Analytical Method

The concentration of each enantiomer, (*R-*) and (*S*)-amlodipine in aqueous phase, was quantified using a Hitachi LC-6200 Intelligent Pump (Tokyo, Japan) for mobile phase delivery to the analytical column. A CHIRAL-AGP analytical column (150 mm x 4.0 mm i.d., particle size 5 μm) (ChromTech, Haegersten, Sweden) was used. Detection was achieved by a Hewlett-Packard 1050 UV Detector (Waldbronn, Germany) at 240 nm. Sample injection was performed via a Rheodyne 7125 Injection Valve (Cotati, California, USA) with a 10 μL loop. A standard curve was used to quantify the enantiomer. The mobile phase was 10 mmol/L acetate buffer solution (pH 4.5): 1-propanol (99:1, v/v) at a flow rate of 0.9 mL/min. The pH of the aqueous phase was measured with a pH meter (Orion, model 720A, USA). All of the above-mentioned chromatographic conditions were adopted from our previous work [27].

## Results and Discussion

### Computational Results

In order to gain insight into the isomers’ differentiation, interaction knowledge between the host and guest molecules is deemed necessary. Computational chemistry was used to calculate the binding energy and to study the 3D optimized structure of the complexes. Table 1 shows the results of the computational calculations. The binding energies of the complexes were calculated as the difference between the energy of the complex and the energies of the free isolated molecules using the PM3 method. The highest negative value of the binding energy is the highest stability of the complex and hence, the strongest interaction between the host and guest molecules.

**Tab. 1:**
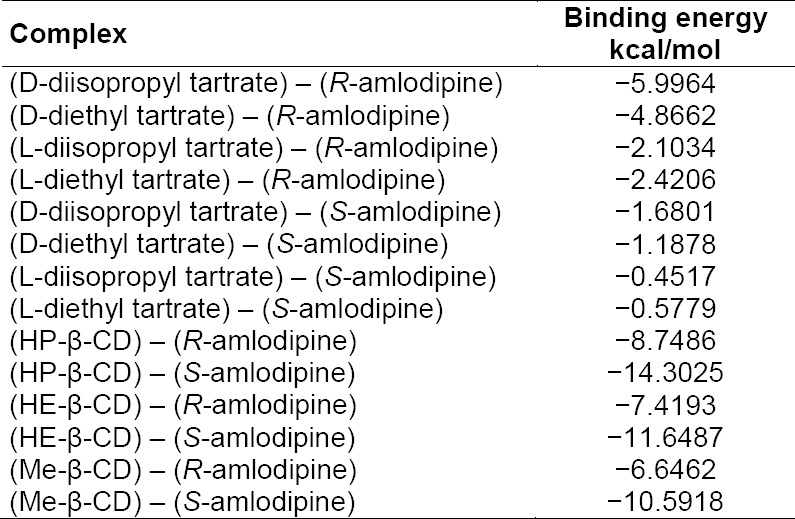
The calculated binding energies of the host-guest complexes using the PM3 semiempirical method

As shown in Table 1, among the different types of the complexes formed between the D- and L- isomers of the tartrate derivatives and the isomers of amlodipine, the complex between D-diisopropyl tartrate and *R*-amlodipine had the highest binding energy with −5.9964 kcal/mol followed by D-diethyl tartrate with *R*-amlodipine (−4.8662 kcal/mol). In the case of the derivatives of β-CD, as a host molecule, the binding energies were higher than those for the tartrate derivatives (Table 1).

**Fig. 2:**
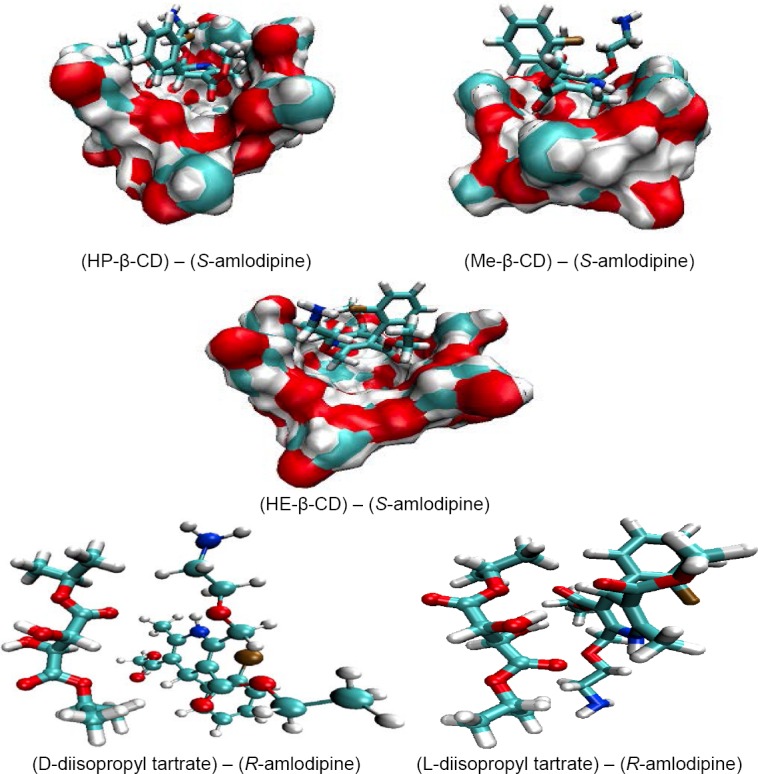
The optimized structures of some host-guest complexes using PM3 calculations

Comparing the binding energies of different types of complexes between β-CD derivatives and *S*-amlodipine, the order was found as follows: HP-β-CD-*S*-amlodipine > HE-β-CD-*S*-amlodipine > Me-β-CD-*S*-amlodipine (Table 1). It can be concluded that β-CD derivatives prefer the *S*-enantiomer. The results of the calculations are in good agreement with the experimental findings. Figure 2 shows the optimized structures for some complexes of β-CD and tartrate derivatives. The interaction forces between the host and guest molecules are hydrogen bonds, electrostatic interactions, and van der Waals, and those forces are found in the case of β-CD and the tartrate derivatives.

### BRCES

The distribution coefficient of *R*- and *S*-amlodipine, *k_R_* and *k_S_*, extracted from the aqueous into the organic phase was determined as prescribed in Eq. 2 and 3:


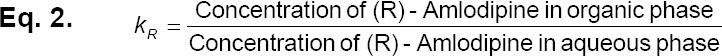



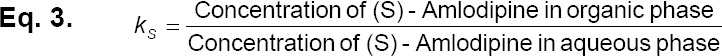


The enantioselectivity or the separation factor (*α*) is defined as the ratio (*k_R_ /k_S_*) of both distribution coefficients of *R*-amlodipine to *S*-amlodipine in an aqueous-organic two-phases system containing a chiral selector in each phase as prescribed by Eq. 4:


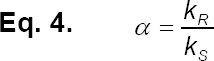


Moreover, α is considered the most important parameter for chiral extraction. For instance, for a 99% pure product (R/S = 100), about 190 NTU (number of transfer units) are required for a separation factor of 1.05; a number decreasing to approximately 30 when α increases to a value of 1.20 [20, 21].

In a biphasic recognition mechanism, formation of diastereomeric complexes between the amlodipine and the tartrate derivative, which is soluble in organic phase, and on the other hand between the amlodipine and the β-cyclodextrin derivative, which is soluble in aqueous phase, occurred due to such molecular interactions as induction, polarization, electrostatics, ligand bond, and hydrogenolysis.

**Fig. 3:**
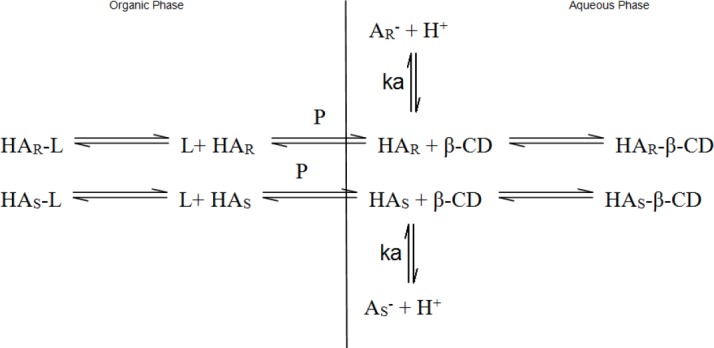
Diagram of the resolution of amlodipine enantiomers by BRCES HA_R_-L…complex of *R*-enantiomer and selector *L* HA_S_-L…complex of *S*-enantiomer and selector *L* L…selector *L* HA_R_-β-CD…complex of *R*-enantiomer and β-CD HA_S_-β-CD…complex of *S*-enantiomer and β-CD k_a_…dissociation constant


Moreover, when the chiral extractants in the organic phase and aqueous phase preferentially recognize *R*-enantiomer and *S*-enantiomer, respectively, then the separation ability of BRCES is greatly improved. Furthermore, it has been reported that the interaction forces in BRCES are much stronger compared to the one present in the monophasic recognition chiral extraction system (MRCES). This is attributed to the cooperation of the forces of both tartrate and β-CD derivatives [47]. In BRCES, the separation of amlodipine enantiomers as hydrophilic β-CD derivatives in aqueous phase preferentially recognizes *S*-amlodipine, whereas in hydrophobic D-diisopropyl tartrate, it is added to the organic phase as a chiral selector, which preferentially recognizes *R*-amlodipine (Fig. 3). Thus, the driving forces for separation of amlodipine enantiomers in BRCES are given by Eq. 5 and 6:

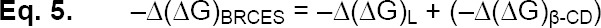



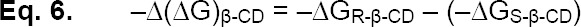


where

As –∆(∆G)_L_ and –∆(∆G)_β-CD_ are all above 0, the driving force –∆(∆G) for separation of amlodipine enantiomers is larger in BRCES than in MRCES. As a result, α-values for BRECES has improved greatly. Therefore, in theory, it can be assumed that BRCES is of stronger separation ability than MRCES.

### Effect of Temperature on Extraction

The influence of temperature on the distribution coefficients of amlodipine enantiomers was investigated over the range of 5–30°C. As observed from Table 2, higher temperature leads to an increase in distribution coefficients as well as a decrease in enantioselectivities. This is attributed to the fact that an increasing distribution coefficients indicates that non-selective physical partitioning increases with temperature while CD complexation decreases. The decrease in enantioselectivities can also be explained by the fact that the selector-enantiomer interaction weakens with temperature and thus the discrimination ability of these selectors for amlodipine enantiomers weakens accordingly.

**Tab. 2:**
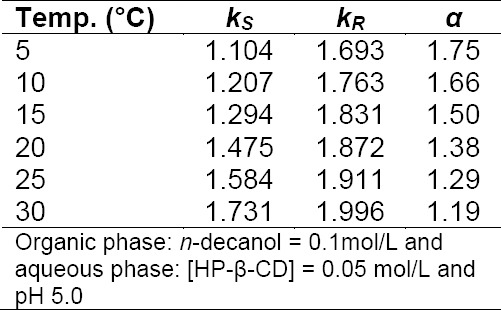
Influence of temperature on the enantioseparation of amlodipine enantiomers

**Fig. 4:**
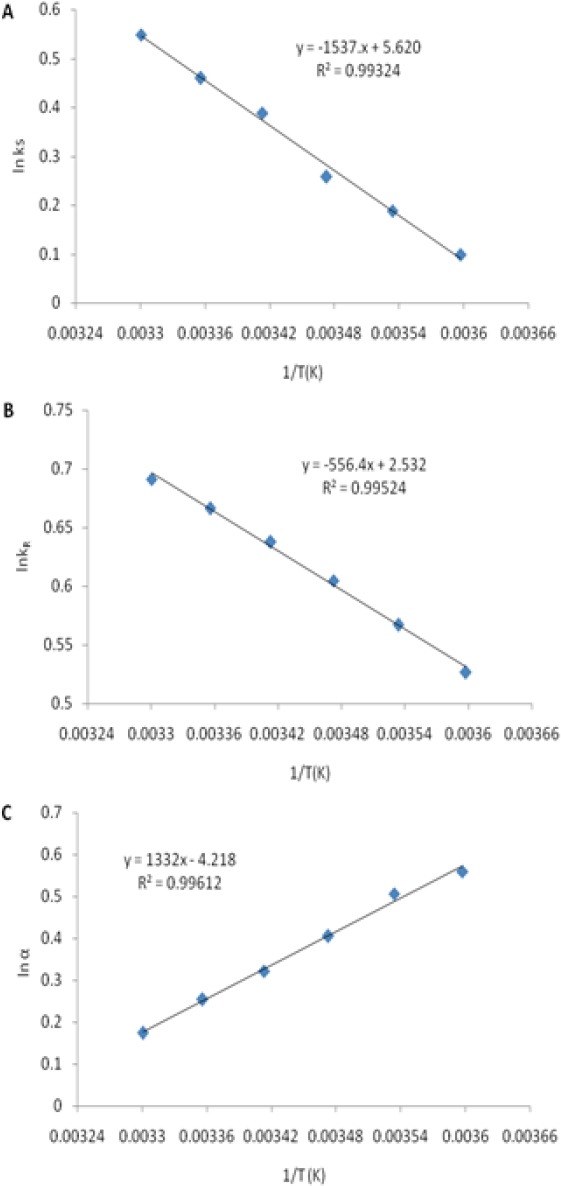
The variations of ln *k* and ln *α* versus 1/*T*

Fig. 4 shows the variations of ln *k* and ln *α* versus 1*/T*. The obtained results fit well with the Van’t Hoff model, indicating that the complexes do not change in conformation and that enantioselective interactions remained unchanged in the temperature range studied [48, 49].

Additionally, the enthalpy change Δ*H* of the extraction process can be calculated from the slopes of the plots of ln *k* versus 1/*T* by Van’t Hoff Eq. 7:


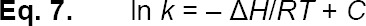


where Δ*H* is the enthalpy change; *R* is the universal gas constant; *C* is a constant. The Δ*H* values were calculated and found to be −15.52 J /mol and −42.88 J /mol for *R*- and *S*-amlodipine, respectively. The different values for the enthalpy change for the two enantiomers may result from the different inclusion ability between HP-β-CD and the two enantiomers as proven by the computational study herein. Moreover, *ΔH* < 0 indicates that the inclusion reaction is an exothermic reaction. This may be attributed to van der Waals interactions and the release of a water molecule with high energy from the ring of the host molecule which results in the negative enthalpy change [50].

### Screening of β-CD and Tartaric Acid Derivatives

The three β-CD derivatives show different enantioselectivities towards amlodipine enantiomers as revealed by the binding energies obtained by computational calculations, where the complexes between the *R-* and *S-*amlodipine pairs and HP-β-CD were the highest binding energies with −8.7486 kcal/mol and −14.3025 kcal/mol, respectively, followed by HE-β-CD and then Me-β-CD (Table 1).

As revealed from our work conducted earlier [27], it shows that HP-β-CD has a higher distribution coefficient and high enantioselectivity when compared to HE-β-CD and Me-β-CD. Moreover, the values of *k_S_* were less than *k_R_* [27], indicating that the three β-CD derivatives recognized the *S*-enantiomer. In other words, the β-CD derivatives formed complexes with *S*-amlodipine and were retained in the aqueous phase which in agreement with the rational computational results obtained. Therefore, HP-β-CD was selected before as a suitable chiral selector in aqueous phase among the three β-CDs derivatives studied.

On the other hand, the values of *k_R_* for D-tartaric acid derivatives were larger than the values of *k_R_* for L-tartaric acid derivatives [27]. This indicates that D-tartaric acid derivatives preferentially recognize *R*-enantiomer. This is also in agreement with the computational study conducted where the D-tartaric acid derivatives formed stable complexes with amlodipine enantiomers rather than with L-tartaric acid derivatives (Table 1). Moreover, it is clear that the enantioselectivities of the extraction increase with the addition of the length of the alkyl chain of D-tartrate [27]. Hence, D-diisopropyl tartrate was chosen as the chiral selector in the organic phase because it has a higher enantioselectivity among the tartaric acid derivatives tested. Therefore, in BRCES for separation of amlodipine enantiomers, D-diisopropyl tartrate and HP-β-CD were chosen previously as the chiral selectors in the organic phase and aqueous phase, respectively. The increase in the distribution coefficients and the enantioselectivities were the results of the mutual aid of HP-β-CD and D-diisopropyl tartrate [22].

## Conclusion

The present theoretical investigation provides a better picture and thus gives more insights into the intermolecular interactions of the inclusion complexes. The inclusion complexation of amlodipine enantiomers with either β-CD or tartaric acid derivatives has been investigated theoretically by performing molecular modeling calculations using the PM3 semiempirical method as well as the docking calculation to complement the experimental studies. The theoretical results revealed the possibility of forming the host–guest inclusion complexes between amlodipine enantiomers and β-CD derivatives. Moreover, in the case of the derivatives of β-CD as a host molecule, the binding energies were higher than those for the tartrate derivatives. Binding energies with different types of β-CD derivatives were as follows: HP-β-CD-*S*-amlodipine > HE-β-CD-*S*-amlodipine > Me-β-CD-*S*-amlodipine, whereas with different types of tartaric acid derivatives, the order was: D-diisopropyl tartrate-*R*-amlodipine > D-diethyl tartrate- *R*-amlodipine > L-diethyl tartrate-*R*-amlodipine > L-diisopropyl tartrate-*R*-amlodipine. It can be concluded that β-CD derivatives prefer the *S*-enantiomer, while D-tartaric acid derivatives prefer the *R*-enantiomer. The complexation between the host and guest molecules is energetically driven by hydrogen bonds, electrostatic interactions, and van der Waals, and those forces are found in the case of β-CD and the tartrate derivatives. The obtained results are considered informative to the relevant experimental research. Moreover, it is found that the separation factors in BRCES are greatly improved due to the mutual aid of the separation forces of tartrate derivatives and HP-β-CD. The computational calculations for the enantioselective partitioning of amlodipine enantiomers rationalized the reasons for the different behavior for such extraction. It can be predicted that liquid-liquid reactive extraction will allow enantioselective separations of a variety of organic compounds on a large-scale.
